# Genome-Wide Aggregated *Trans*-Effects Analysis Implicates Deficient Type III Interferon Signaling as a Key Cause of Inflammatory Bowel Disease

**DOI:** 10.1093/ibd/izaf214

**Published:** 2025-09-25

**Authors:** Paul M McKeigue, Andrii Iakovliev, Buddhiprabha Erabadda, Helen M Colhoun, Athina Spiliopoulou

**Affiliations:** Usher Institute, College of Medicine and Veterinary Medicine, University of Edinburgh, Edinburgh, Scotland; Institute of Genetics and Cancer, College of Medicine and Veterinary Medicine, University of Edinburgh Western General Hospital campus, Edinburgh, Scotland; Institute of Genetics and Cancer, College of Medicine and Veterinary Medicine, University of Edinburgh Western General Hospital campus, Edinburgh, Scotland; Usher Institute, College of Medicine and Veterinary Medicine, University of Edinburgh, Edinburgh, Scotland; Institute of Genetics and Cancer, College of Medicine and Veterinary Medicine, University of Edinburgh Western General Hospital campus, Edinburgh, Scotland; Usher Institute, College of Medicine and Veterinary Medicine, University of Edinburgh, Edinburgh, Scotland; Institute of Genetics and Cancer, College of Medicine and Veterinary Medicine, University of Edinburgh Western General Hospital campus, Edinburgh, Scotland

**Keywords:** quantitative trait locus, interferon-stimulated genes, genetics

## Abstract

**Background:**

Genome-wide association studies of inflammatory bowel disease have identified hundreds of common genetic variants that are associated with inflammatory bowel disease, but few promising therapeutic targets. The “omnigenic” sparse effector hypothesis postulates that the polygenic effects of common SNPs on a typical complex trait are mediated by *trans*-effects that coalesce on the expression of a sparse set of core genes. The objective of this study was to identify core genes for inflammatory bowel disease.

**Methods:**

Using summary statistics from studies of transcript levels in whole blood or proteins in plasma, we constructed genome-wide aggregated *trans*-effects (GATE) scores for predicted gene expression in the UK Biobank cohort and tested these scores for association with inflammatory bowel disease (7949 cases, 452 790 noncases).

**Results:**

Inflammatory bowel disease was inversely associated with GATE scores for 5 interferon-stimulated genes—*IFIT1, IFI44, HERC5, MX1, IFI44L—*regulated by the same *trans*-expression quantitative trait locus, and with the GATE score for *IFNL1*. For 6 other genes, GATE score associations with inflammatory bowel disease were supported by other criteria: reported associations with nearby genetic variants, perturbation in experimental models, association with measured protein levels, or drug effects.

**Conclusions:**

These results implicate down-regulation of Type III interferon signaling as a core pathway in the etiology of inflammatory bowel disease, supported by reports of monogenic inflammatory bowel disease caused by rare loss-of-function variants and by perturbation in experimental models of colitis. Deficient Type III interferon signaling may be amenable to therapeutic intervention.

Key Messages What is already known?

Inflammatory bowel disease is under strong genetic influence, but genetic studies have failed to identify the key therapeutic targets.

What is new here?

This study uses a novel method of genetic analysis that aggregates predicted *trans*-effects of common variants to identify genes and pathways on which these effects coalesce to cause inflammatory bowel disease; the results implicate deficient Type III interferon signaling as a core pathway in the etiology of inflammatory bowel disease.

How can this study help patient care?

Deficient Type III interferon signaling may be amenable to therapeutic intervention, for instance, by engineered probiotics or nutritional supplements.

## Introduction

Inflammatory bowel disease, in the form of ulcerative colitis or Crohn’s disease, affects about 0.6% of the population. Immunological and genetic studies indicate that dysregulation of both innate and adaptive immune systems contributes to the inflammatory response. Although the GWAS Catalog lists 274 genomic regions outside the human leukocyte antigen (HLA) region that contain SNPs associated with inflammatory bowel disease at the conventional threshold of *P < *5 × 10^−8^, the genes in these regions are mostly broadly expressed and are not in pathways specifically relevant to inflammation.^1^ The “omnigenic” sparse effector model was proposed as a fundamental rethink of the genetic architecture of complex traits.^2^^,^^3^ This postulates that most of the polygenic effects on a typical complex trait are mediated through weak *trans*-effects of common variants that coalesce on the expression of a relatively sparse set of “core” effector genes in relevant tissues. Because disease-relevant genes are enriched with redundant enhancer domains and depleted of *cis*-expression quantitative trait locus (eQTLs) of large effect, they are not easily detected in a conventional SNP-by-SNP GWAS.^4^^,^^5^

The availability of summary statistics from large genome-wide association studies of transcripts in whole blood or proteins in plasma has made it possible to test this hypothesis by constructing genotypic predictors of gene expression based on aggregated *trans*-effects, and testing these genotypic scores for association with the disease or trait under study. Although these genotypic scores are only weakly correlated with measured gene expression, they can be used to infer causal effects of the target genes on the disease if the effects of these genes are large. We have reported application of this genome-wide aggregated *trans*-effects (GATE) analysis pipeline to type 1 diabetes and to rheumatoid arthritis in which we identified *CTLA4*, *PDCD1*, and other genes encoding immune checkpoints as core genes for these autoimmune diseases.^6–8^ The objective of this study was to investigate whether the same approach can identify core genes (defined as effector genes that directly influence disease risk) or core pathways (defined as pathways on which *trans*-effects of common variants coalesce to cause disease possibly through regulation of effector genes) for inflammatory bowel disease.

## Materials and Methods

### Case definition

The case definition of inflammatory bowel disease in the UK Biobank cohort was based on either of 2 criteria:

- a hospital discharge or death certificate with an International Classification of Diseases diagnostic code for ulcerative colitis or Crohn’s disease.
- a self-reported or primary care diagnosis of inflammatory bowel disease, supported by prescription of a relevant drug—5-aminosalicylates, prednisolone, beclomethasone, hydrocortisone, methotrexate, or mercaptopurine—at baseline or during follow-up.

Of 487 044 individuals with nonmissing phenotype and genotype data, 8329 had ever been diagnosed with inflammatory bowel disease. The full dataset was pruned to ensure that no pairs of individuals with a kinship coefficient greater than 0.05 remained. As *cis*- and *trans*-effects of SNP genotypes were estimated from studies of individuals of European ancestry, the dataset was restricted to those classified as European by a *k*-nearest neighbor algorithm using the first 10 genetic principal components. After applying these exclusions, there were 460 699 individuals of whom 7949 were classified as cases.

### GATE analysis

Methods for GATE analysis have been described previously.^6^^,^^8^ In the first step, summary statistics from a genome-wide association study (GWAS) of levels of transcripts or circulating proteins are used to compute predicted *trans*-effects on the expression of each gene from the genotypes of each individual in the target cohort, and aggregated into a GATE score for predicted expression of each gene in each individual. In the second step, scores for the expression of each gene are tested for association with the disease or trait under study.

To construct GATE scores, the list of SNPs that were typed or imputed in the case-control dataset is uploaded to the GENOSCORES server. For each target gene, a database query extracts the univariate coefficients of regression of gene expression on each of these SNPs, filtered at *P < *10^−5^. Each chunk of SNPs that contains at least one SNP association at *P < *10^−6^ and is separated by at least 1 Mb from other such chunks is assigned as an eQTL. The eQTL is classified as *cis*- if the distance from the chunk to the transcription site of the respective gene is less than or equal to 5 Mb, and as *trans*- if the distance is more than 5 Mb. For each eQTL, the vector of univariate coefficients is premultiplied by the inverse correlation matrix between SNP genotypes (obtained from the 1000 Genomes reference panel) to obtain a vector of multivariable weights that are corrected for linkage disequilibrium. This adjustment approximates the regression coefficients that would be obtained in a multiple regression analysis of the individual-level GWAS dataset. The locus-specific score is calculated for each individual as the dot product of the individual’s genotypes and the adjusted weights vector. The locus-specific scores are summed over *trans*-eQTLs to obtain the GATE score for each individual. *Cis*-eQTLs are excluded from the aggregated *trans*-scores and tested separately for association with the disease. The procedure for protein QTLs (pQTLs) is the same as that for eQTLs.

*Trans*-eQTL scores were computed from eQTLGen Phase 1, in which only 10 317 trait-associated SNPs were tested for *trans*-associations.^9^  *Trans*-pQTL scores were computed from 2 studies of circulating proteins:

- 4719 proteins on the SomaLogic v4 panel were measured in plasma on 35 559 Icelanders in the DeCODE study.^10^ 2207 aptamers on this platform that appeared to cross-react with complement factor H were excluded. The criteria for identifying these aptamers were: a *trans*-pQTL at the *CFH* locus, no *cis*-pQTL, and the *trans*-pQTL was associated with age-related macular degeneration.
- 2923 proteins on the Olink Explore panel measured in plasma on 49 235 participants of European ancestry in the UK Biobank.^11^

As the HLA region is a hotspot for *trans*-eQTLs for genes involved in immunity and inflammation,^12^ and associations of these *trans*-eQTLs with disease are heavily confounded by the direct effects of HLA antigens, the HLA region (from 25 to 34 Mb on chromosome 6) was excluded from the computation of genome-wide *trans*-scores, as previously described.^6^

### Statistical analysis

When testing for association with genotypic scores for Olink proteins calculated from the UK Biobank proteomics study, the 54 306 participants who were included in the proteomics study were excluded from tests of association of the genotypic scores with the outcome. A logistic regression model was fitted with inflammatory bowel disease as the response variable with sex and the first 20 genetic principal components as covariates. The fitted values from this null model were used to compute tests for association with the *cis*-score and aggregated *trans*-score for each transcript or circulating protein, based on the gradient (efficient score) and negative second derivative (Fisher information) of the log-likelihood at the null. Log odds ratios in the tables have been standardized so that the coefficient is the log odds ratio associated with an increase of the *trans*-score, *cis*-score, or measured protein level by one standard deviation.

As an index of the effective number of unlinked *trans*-eQTLs contributing to each GATE score, we calculated the diversity index or Hill number.^13^ For each gene, the diversity index was computed from the variances *σ*_1_*,…,σ_K_* of the *K* locus-specific *trans* scores as 2^−∑^  *^p^_i_*  ^log^  _2 _*^p^_i_*, where *p_i_* = *σ_i_*^2^∕∑ *σ_i_*^2^. This index can take values from 1, if one of the eQTLs has a much larger variance than the others, to *K*, if the variances of the locus-specific *trans*-scores are equal.

### Filtering and validation criteria

As previously,^6^ the GATE scores were filtered to retain only those for which the effective number of *trans*-QTLs was greater than 5. This increases the ratio of “signal” (causal effects consistent in direction) to “noise” (direct effects of genotype on outcome, assumed to be random in direction). This filtering retained 413 aggregated eQTL scores for 413 unique genes and 3256 aggregated pQTL scores for 2586 unique genes. In this analysis, the number of independent tests is effectively the number of genes for which a GATE score based on at least 5 effective *trans*-QTLs can be computed. This prior hypothesis space is far smaller than in a conventional GWAS study that tests millions of SNPs. On this basis, we set a threshold of *P < *10^−5^ for initial filtering of associations of disease with GATE scores. Where there was a reported GWAS association with inflammatory bowel disease within 200 kb of the transcription site of the gene, GATE scores associated with inflammatory bowel disease at *P < *10^−4^ were included. These possible core genes were subjected to further validation tests as described below.

Association of a GATE score for a gene with disease may be confounded by direct effects of genetic variants on the disease that are not mediated through the gene under study. To evaluate whether GATE score associations with disease are likely to be causal, we used 3 criteria based on *trans*-QTLs alone:

1. Effective number of *trans*-QTLs contributing to the GATE score: if many *trans*-QTLs contribute to the aggregated score, it is less likely that a few “peripheral master regulators” can account for the association of the score with disease.
2. *Trans*-pQTLs contributing to the GATE score are not shared with disease-associated GATE scores for other genes. To assess this visually, we computed correlations between GATE scores that were associated with disease, reordered the rows and columns of the correlation matrix to make it as nearly as possible block-diagonal, and examined a heatmap of this matrix to identify blocks of GATE scores with pleiotropic effects on gene expression. For a more detailed examination, we grouped *trans*-QTLs that contributed to disease-associated GATE scores into overlapping clumps and tabulated the genes regulated in *trans* by these clumps.
3. Dose-response relationship between the effects of the *trans*-QTLs on the expression of the gene and the effects of these *trans*-QTLs on the disease. The method of 2-sample Mendelian randomization analysis is effectively a test for a dose-response relationship. The test for causality depends on the assumption that the direct effects of the genetic instruments on the disease and the effects of the instruments on the transcript levels are not coupled by a shared pathway; such coupling may identify a causal pathway even if the target gene is not itself causal. We have shown elsewhere that the statistical power of a 2-sample Mendelian randomization analysis to detect a causal effect depends critically upon the number of genetic instruments: in this case, the number of *trans*-QTLs. For this reason, we restricted this analysis to genes with at least 10 *trans*-QTLs. We tested for causality by marginalizing over the distribution of (unobserved) direct effects to compute the likelihood of the causal effect parameter given the data. This is similar to a Bayesian method described previously^14^ except that our model includes a parameter that regularizes the size of the nonzero direct effects,^15^ the method is implemented in the Stan probabilistic programming language, and we divide the posterior distribution of the causal effect parameter by the prior to obtain the marginal likelihood, from which the maximum likelihood estimate and *P*-value are calculated.

For each putative core gene, we evaluated 5 other criteria for causality that do not depend on *trans*-QTLs:

1. Monogenic disease is caused by rare variants in the gene or in other genes in the same pathway.
2. Association of disease with common variants within 200 kb of the transcription site of the gene, at conventional levels of genome-wide significance. As *cis*-SNPs (within 5 Mb of the transcription site) were excluded from GATE scores, any association of disease with SNPs near the transcription site is independent validation of an effect of the gene.
3. Association of disease with measured levels of the protein. This can be evaluated only for proteins on the Olink panel that were measured in the UK Biobank proteomics study. Because GATE scores typically explain only a small proportion of the variance of the measured protein, the association of disease with the measured protein should be far stronger than the association with the GATE score if the association with the protein is causal.
4. Experimental evidence that perturbing the gene by knockout, transduction, or over-expression, or perturbation of the gene product by an inhibitor or an agonist, alters the severity of disease in an experimental model.
5. Drugs targeting the gene product, its ligand, or its receptor cause the disease, or have shown efficacy against the disease in a clinical trial.

## Results

### Associations of aggregated *trans*-QTL scores with inflammatory bowel disease

**Figure 1.**
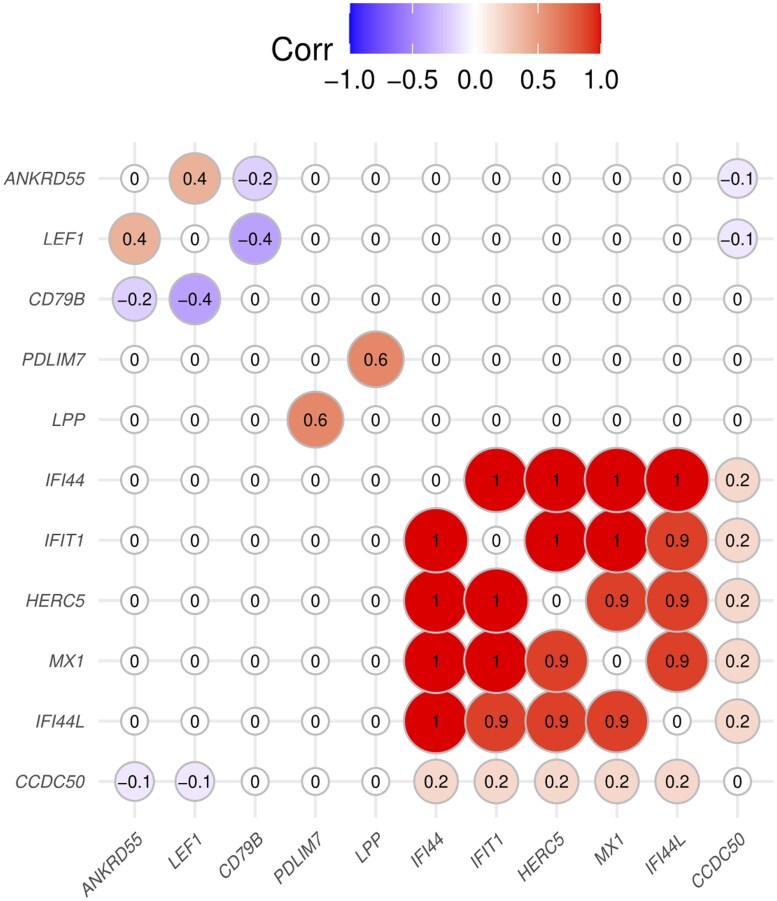
Correlations between aggregated eQTL or pQTL scores for putative core genes identified in Table 1. Rows and columns of the correlation matrix are ordered by hierarchical clustering on the absolute value of correlation. eQTL, expression quantitative trait locus; pQTL, protein quantitative trait locus.

Table 1 shows the GATE scores associated with inflammatory bowel disease, after filtering by effective number of *trans*-QTLs: 9 through association with aggregated *trans*-eQTL scores, and 2 through association with aggregated *trans*-pQTL scores. Figure 1 shows that the aggregated *trans*-eQTL scores for 5 of these genes—*IFIT1*, *IFI44*, *HERC5*, *MX1*, *IFI44L—*are highly correlated, indicating that they share the same *trans*-eQTLs. These genes are recognizable as interferon-stimulated genes (ISGs) encoding proteins that inhibit viral replication: their co-expression is often termed an “interferon signature”. We sought replication of this result in summary statistics for “expression quantitative trait analysis” previously released by the eQTLGen Consortium.^9^ Their approach identifies core genes by testing for the association of polygenic risk scores for the disease with measured levels of expression of each gene.^16^  Table S1 shows that measured levels of all 5 of the ISGs in Table 1 were inversely associated with polygenic scores for Crohn’s disease or ulcerative colitis, calculated from a meta-analysis that did not include the UK Biobank cohort.^17^  Table S1 also shows for these 5 genes estimates for the SNP heritability of transcript levels in whole blood, partitioned into *cis*- and *trans*- components, extracted from a study of twins.^18^ The heritability attributable to *trans*-effects varies from 35% to 41% with very little heritability attributable to *cis*-effects except for *MX1*.

**Table 1.** Putative core genes identified through aggregated *trans*-QTL scores.

		Transcription site			*trans*-score			*cis*-score	
QTL study	Gene	Chrom	Start position (Mb)	Reported GWAS hit within 200 kb	Effective number of *trans*-eQTLs	Log odds ratio	*P*-value	Log odds ratio	*P*-value
**eQTL scores (413 genes)**									
eQTLGen	*LEF1*	4	108.05	.	6.8	0.057	7 × 10^ − 7^	0.007	.5
eQTLGen	*ANKRD55*	5	56.10	*IL6ST, IL31RA, ANKRD55*	7.6	0.052	8 × 10^ − 6^	0.008	.5
eQTLGen	*CD79B*	17	63.93	*ERN1*	9.5	−0.052	8 × 10^ − 6^	−0.009	.4
eQTLGen	(*IFIT1*)	10	89.39	.	5.4	−0.054	2 × 10^ − 6^	−0.005	.6
eQTLGen	(*IFI44*)	1	78.65	.	5.3	−0.057	4 × 10^ − 7^	−0.027	.02
eQTLGen	(*HERC5*)	4	88.46	.	5.2	−0.057	4 × 10^ − 7^	.	.
eQTLGen	(*MX1*)	21	41.42	.	6.2	−0.054	2 × 10^ − 6^	0.000	1
eQTLGen	(*IFI44L*)	1	78.62	.	6.1	−0.057	4 × 10^ − 7^	−0.002	.9
eQTLGen	*CCDC50*	3	191.33	.	5.4	−0.060	3 × 10^ − 7^	0.011	.3
**pQTL scores (2586 genes)**									
UKB	*LPP*	3	188.15	*LPP*	10.2	−0.051	3 × 10^ − 5^	.	.
UKB	*PDLIM7*	5	177.48	*RGS14, SLC34A1, DOK3*	8.5	−0.050	4 × 10^ − 5^	−0.019	.1

Genes are ordered by hierarchical clustering as in Figure 1. Genes with aggregated *trans*-scores that are highly correlated (absolute value > 0.8) with the score for another gene are shown with the gene symbol in parentheses.

Abbreviations: eQTL, expression quantitative trait locus; pQTL, protein quantitative trait locus; QTL, quantitative trait locus.

Table 2 shows the *trans*-eQTLs shared by the 5 ISGs. The *trans*-eQTLs contributing to this association with GATE scores for ISGs include *IFIH1*, *IKZF1,* and *CARD9*, previously reported as GWAS hits for inflammatory bowel disease. *IFIH1* encodes MDA-5, a cytosolic sensor of dsRNA that initiates interferon signaling. *IKZF1* regulates the development and function of plasmacytoid dendritic cells^19^; this cell type has a specialized capacity for production of Type I and Type III interferons.^20^  *CARD9* mediates the activation by C-type lectin receptors of nuclear factor *κ*B,^21^ which regulates a subset of ISGs.^22^

**Table 2.** *Trans*-eQTLs for interferon-stimulated genes in Table 1.

Chrom	Clump start position (Mb)	Clump end position (Mb)	Target genes	Genes in or near *trans*-pQTL clump
1	183.57	183.57	*HERC5, IFIT1, MX1*	*NCF2, SMG7*
2	162.25	162.40	*HERC5, IFI44, IFI44L, IFIT1, MX1*	*GCA,* ***IFIH1*** *, KCNH7*
3	159.92	159.95	*IFI44L, MX1*	*IL12A-AS1*
7	50.22	50.33	*CCDC50, IFIT1, MX1, HERC5, IFI44, IFI44L*	***IKZF1***
9	32.43	32.52	*HERC5, IFI44, IFI44L, IFIT1, MX1*	*ACO1, RIGI*
9	136.36	136.46	*HERC5, IFI44, IFI44L, IFIT1, MX1*	***CARD9*** *,* ***DNLZ*** *, ENTR1,* ***GPSM1*** *,* ***INPP5E*** *,* ***PMPCA*** *,* ***SEC16A*** *,* ***SNAPC4***
10	48.82	48.91	*HERC5, IFI44, IFI44L, IFIT1, MX1*	*WDFY4*
12	111.27	112.43	*ANKRD55, CCDC50, CD79B, LEF1, IFI44, IFI44L*	***ACAD10*** *, ADAM1A, ADAM1B,* ***ALDH2*** *,* ***ATXN2*** *, ATXN2-AS,* ***BRAP*** *, CUX2,* ***ERP29*** *, HECTD4,* ***MAPKAPK5*** *,* ***NAA25*** *,* ***SH2B3*** *,* ***TMEM116*** *, …*
15	51.23	51.46	*MX1, HERC5, IFI44, IFI44L, IFIT1*	*CYP19A1, DMXL2, GLDN, MIR4713, MIR4713HG, …*
16	31.27	31.36	*HERC5, IFI44, IFI44L*	*ITGAM*
17	46.71	46.78	*MX1, HERC5, IFI44, IFI44L, IFIT1*	*NSF, RPS7P11, WNT3*

Each row is a clump of *trans*-pQTLs for an interferon-stimulated target gene. In each clump, genes previously reported as GWAS hits are shown in bold, together with up to 5 genes not reported as GWAS hits.

Abbreviations: eQTL, expression quantitative trait locus; pQTL, protein quantitative trait locus.

As the GATE scores for the 5 ISGs are highly correlated and share the same *trans*-eQTLs, these results do not identify a specific causal role for any one of these genes; rather, they point to interferon signaling as a shared pathway on which common genetic variants coalesce to influence risk of inflammatory bowel disease.

**Figure 2.**
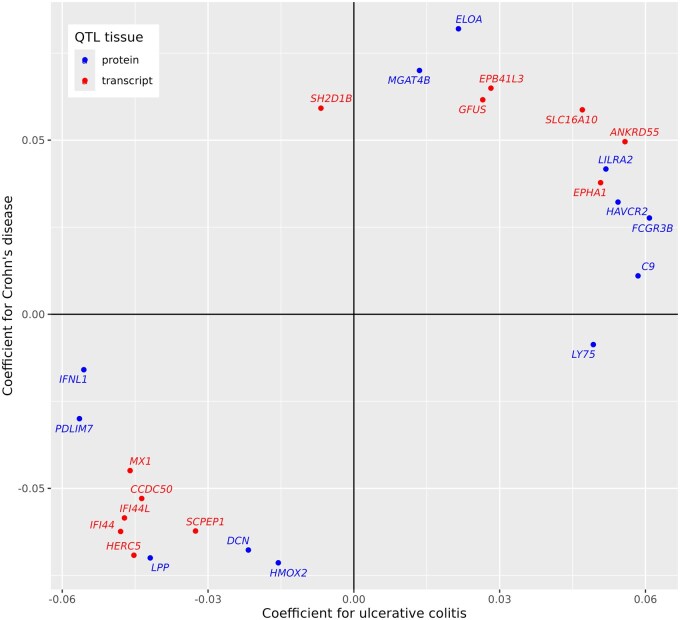
Comparison of GATE score associations with ulcerative colitis and Crohn’s disease, tested separately. Coefficients with *P ≤ *5 × 10^ − 4^ for association with either phenotype are shown. GATE, genome-wide aggregated *trans*-effects.

This led us to look more closely at associations of inflammatory bowel disease with GATE scores for genes that encode interferons or interferon receptors, and with measured levels of the proteins encoded by these genes. Table 3 shows the associations of inflammatory bowel disease with measured plasma levels of interferons or soluble isoforms of interferon receptors in the UK Biobank proteomics study, and with the GATE scores for these proteins. The GATE score for *IFNL1*, which encodes interferon-*λ*1, was inversely associated with inflammatory bowel disease, consistent with the direction of association of GATE scores for ISGs. Figure 2 shows that this association was strongest for ulcerative colitis (standardized log odds ratio −0.30, *P = *6* × *10^−5^).

**Table 3.** Associations of inflammatory bowel disease with plasma levels of interferons or soluble interferon receptors measured in the UK Biobank proteomics study, and with GATE scores for these proteins in UK Biobank participants who were not in the proteomics study.

		Transcription site			Measured protein levels				GATE score			
Gene symbol	Gene name	Chr	Start position (Mb)	Reported GWAS hit within 200 kb	Non-cases	Cases	Log odds ratio	*P*-value	*r* ^2^	Effective number of *trans*-QTLs	Log odds ratio	*P*-value
*IFNLR1*	Interferon lambda receptor 1	1	24.2	*IFNLR1*	49 734	921	0.09	.006	0.007	8.6	0.02	.9
*IFNGR1*	Interferon gamma receptor 1	6	137.2	.	50 017	932	0.20	1 × 10^ − 9^	0.024	17.2	0.03	.5
*IFNW1*	Interferon omega 1	9	21.1	.	41 826	762	0.01	.8	0.001	3.0	.	.
*IFNG*	Interferon gamma	12	68.2	*IFNG, IL26, IL22, IFNG-AS1*	48 889	902	0.18	8 × 10^ − 8^	0.008	10.2	.	.
*IFNL2*	Interferon lambda 2	19	39.3	.	41 861	766	0.04	.2	0.002	3.9	.	.
*IFNL1*	Interferon lambda 1	19	39.3	.	48 652	900	0.04	.3	0.016	5.9	−0.22	6 × 10^ − 4^
*IFNAR1*	Interferon alpha and beta receptor subunit 1	21	33.3	*IFNGR2, IFNAR1, IFNAR2, IL10RB, GART, TMEM50B*	43 068	791	−0.04	.3	0.008	4.5	.	.
*IFNGR2*	Interferon gamma receptor 2	21	33.4	*IFNGR2, IFNAR1, IFNAR2, IL10RB, GART, TMEM50B*	49 427	914	0.04	.2	0.015	16.2	0.03	.6

Abbreviations: GATE, genome-wide aggregated *trans*-effects; QTL, quantitative trait locus.

The GATE scores for the other 6 genes in Table 1 are not highly correlated with GATE scores for ISGs. Table S2 shows the results of Mendelian randomization analysis for the 5 genes that had at least 10 *trans*-QTLs. For *ANKRD55* and *VCAM1*, there is a dose-response relationship supporting causality. For *ANKRD55*, the associations of inflammatory bowel disease with GATE scores were replicated by associations of a polygenic score for ulcerative colitis with measured expression levels, as shown in Table S1. Three of these genes—*ANKRD55*, *LPP*, *PDLIM7—*were supported by a reported GWAS hit within 200 kb of the transcription site, and one—*CCDC50—*by experimental validation in a mouse model.^23^  Figure 2 compares the associations of GATE scores with ulcerative colitis with the associations of the same scores with Crohn’s disease. For the GATE scores in Table 1, associations with these 2 subtypes of inflammatory bowel disease were mostly similar in magnitude and direction.

### Associations with circulating levels of proteins

The UK Biobank (UKBB) proteomics study included 947 cases and 51 647 noncases of inflammatory bowel disease. In logistic regression models adjusted for age and sex, and genetic ancestry, 192 Olink proteins were associated with inflammatory bowel disease at *P < *10^−9^. Table 4 shows that for 2 of these proteins—encoded by *IL6* and *VCAM1—*there was genetic support for causality of the protein in the form of a GATE score association with the disease in the same direction. For *VCAM1*, there is additional support for causality in an experimental model^24^ and from the effects of drugs targeting its ligand α4β7.^25^ For *IL6*,there is support from the effect of a selective inhibitor of IL-6 signaling in a clinical trial.^26^  Table 4 also shows that for another 40 of these proteins associated with inflammatory bowel disease, there was genetic support for causality from a nearby GWAS hit.

**Table 4.** Proteins associated with inflammatory bowel disease at *P < *10^−9^ that have genetic support either from GATE score associations with disease at *P < .*001 or from reported GWAS association of nearby SNPs with disease.

				Measured level of protein				GATE score			
Gene encoding protein	Chr	Start position (Mb)	GWAS hit within 200 kb	Non-cases	Cases	Log odds ratio	*P*-value	Effective number of *trans*-QTLs	*r* ^2^	Log odds ratio	*P*-value
**Genetic support from GATE scores**											
*VCAM1*	1	100.72	.	49 914	926	0.21	2 × 10^ − 10^	17.2	0.044	0.05	6 × 10^ − 5^
*IL6*	7	22.73	.	49 569	926	0.29	1 × 10 ^− 21^	4.5	0.004	0.05	1 × 10^ − 4^
**Genetic support from GWAS hit within 200 kb**											
*TNFRSF14*	1	2.56	*TNFRSF14*	49 851	930	0.28	5 × 10^ − 17^	9.8	0.010	0.00	1
*TNFRSF9*	1	7.92	*TNFRSF9*	49 427	914	0.24	4 × 10^ − 13^	9.0	0.017	0.00	.7
*REG4*	1	119.79	*ADAM30*	49 851	930	0.32	2 × 10 ^− 21^	18.4	0.016	0.01	.5
*EFNA4*	1	155.06	*PMVK, PBXIP1*	49 345	912	0.22	4 × 10^ − 11^	10.2	0.013	0.02	.08
*EFNA1*	1	155.13	*PMVK, PBXIP1*	49 729	928	0.25	3 × 10^ − 14^	13.6	0.009	−0.01	.5
*SLAMF8*	1	159.83	*SLAMF8*	48 653	895	0.22	3 × 10 ^− 11^	5.7	0.007	0.01	.3
*NECTIN4*	1	161.07	*ITLN1, ITLN2*	49 949	932	0.21	8 × 10^ − 12^	17.7	0.019	0.00	.7
*IL10*	1	206.77	*IL10*	48 889	902	0.26	1 × 10^ − 19^	4.2	0.010	−0.01	.3
*IL19*	1	206.77	*IL19*	48 597	902	0.27	3 × 10 ^− 17^	2.5	0.041	0.03	.03
*IL1R1*	2	102.06	*IL1R1*	49 729	928	0.23	3 × 10^ − 12^	13.4	0.038	−0.01	.5
*IL18R1*	2	102.31	*IL18R1*	50 338	929	0.22	5 × 10^ − 11^	17.1	0.022	0.00	.9
*ITGAV*	2	186.59	*ITGAV*	50 210	933	-0.21	6 × 10 ^− 10^	13.5	0.016	0.01	.3
*CCL20*	2	227.81	*CCL20*	50 338	929	0.31	7 × 10^ − 25^	7.0	0.011	0.00	.9
*PDCD1*	2	241.85	*PDCD1*	50 126	932	0.23	2 × 10^ − 12^	21.3	0.034	0.01	.3
*CD74*	5	150.40	*SLC6A7, CAMK2A*	50 233	928	0.29	9 × 10^ − 17^	4.4	0.021	0.02	.08
*IL12B*	5	159.31	*IL12B*	50 338	929	0.22	4 × 10^ − 11^	18.4	0.075	0.00	.8
*RNASET2*	6	166.92	*RNASET2*	50 175	925	0.22	4 × 10^ − 11^	7.1	0.024	0.03	.01
*CD274*	9	5.45	*JAK2*	49 427	914	0.29	5 × 10^ − 18^	8.3	0.017	−0.02	.1
*TNC*	9	115.02	*TNC*	49 995	928	0.26	8 × 10^ − 15^	8.8	0.078	0.01	.2
*IL2RA*	10	6.01	*IL2RA*	49 470	905	0.33	3 × 10 ^− 23^	11.6	0.016	0.02	.04
*FAS*	10	88.95	*ACTA2*	50 247	926	0.21	1 × 10^ − 11^	5.6	0.004	0.01	.5
*PRG3*	11	57.38	*PRG3*	43 253	795	0.27	2 × 10^ − 14^	31.1	0.069	0.01	.3
*PRG2*	11	57.39	*PRG2*	43 253	795	0.35	1 × 10 ^− 22^	27.8	0.076	0.02	.09
*CD5*	11	61.10	*CD5*	50 136	933	0.24	6 × 10 ^− 14^	11.3	0.022	0.02	.1
*TNFRSF1A*	12	6.33	*TNFRSF1A*	49 813	929	0.30	1 × 10^ − 18^	14.3	0.021	0.00	.7
*CD27*	12	6.44	*CD27*	49 949	932	0.26	8 × 10^ − 16^	24.2	0.021	0.01	.3
*MANSC1*	12	12.33	*LOH12CR1*	49 949	932	0.21	6 × 10^ − 11^	41.5	0.055	−0.01	.4
*IL22*	12	68.25	*IL22*	43 046	791	0.37	3 × 10^ − 26^	6.2	0.005	0.03	.03
*PRSS8*	16	31.13	*SETD1A*	49 610	927	0.24	4 × 10^ − 11^	11.6	0.013	0.01	.3
*LGALS9*	17	27.63	*LGALS9*	49 779	927	0.24	4 × 10 ^− 12^	8.6	0.015	0.04	.004
*NOS2*	17	27.76	*NOS2*	41 826	762	0.39	4 × 10^ − 38^	3.9	0.004	0.04	.001
*CCL7*	17	34.27	*CCL7*	49 734	921	0.22	8 × 10^ − 11^	4.9	0.023	0.00	.8
*CCL11*	17	34.29	*CCL11*	50 338	929	0.33	4 × 10^ − 22^	11.4	0.022	0.00	.9
*CD79B*	17	63.93	*ERN1*	50 338	929	0.22	1 × 10^ − 11^	15.9	0.034	−0.01	.7
*FCAR*	19	54.87	*KIR2DL1, LILRB4*	49 734	921	0.21	2 × 10^ − 10^	15.1	0.035	−0.01	.3
*MMP9*	20	46.01	*MMP9*	49 731	928	0.21	4 × 10^ − 10^	10.6	0.031	−0.03	.02
*TNFRSF6B*	20	63.70	*TNFRSF6B*	49 427	914	0.31	1 × 10^ − 21^	6.5	0.008	0.01	.2
*IL10RB*	21	33.27	*IL10RB*	50 017	932	0.22	1 × 10^ − 10^	20.8	0.015	0.02	.1
*TFF3*	21	42.31	*UBASH3A*	49 750	924	0.20	5 × 10^ − 11^	2.0	0.008	−0.02	.05
*OSM*	22	30.26	*OSM*	49 522	917	0.27	1 × 10^ − 15^	9.3	0.032	−0.01	.6

Associations are adjusted for age, sex, and continental ancestry. GATE score associations are with the scores computed from UK Biobank summary statistics. Proteins encoded by genes in the HLA region are excluded.

Abbreviations: GATE, genome-wide aggregated *trans*-effects; QTL, quantitative trait locus.

## Discussion

Though the original objective of this study was to identify core genes for inflammatory bowel disease, an unexpected result was the identification of interferon signaling as a core pathway through which multiple common variants increase the risk of inflammatory bowel disease by downregulating interferon signaling, and thus reducing the expression of multiple ISGs that constitute an interferon signature when co-expressed. The association of inflammatory bowel disease with genetic variants that downregulate the expression of these genes was replicated by the results of expression quantitative trait analysis, which tests for the association of measured expression levels of each gene with polygenic scores for the disease.^9^ These polygenic scores were learned from a meta-analysis of case-control studies^17^ that does not overlap with the UK Biobank cohort. Expression quantitative trait analysis may have less statistical power than GATE analysis because aggregating the effects of disease-associated SNPs into a polygenic score dilutes the association of *trans*-eQTLs with the gene under study.

Studies of the mechanisms underlying *trans*-eQTLs have focused on signaling mechanisms within cells, including transcription factors and protein-protein interactions.^9^ This study shows, however, that *trans*-eQTLs can be generated by signaling between cells of different types; thus, SNPs in *IFIH1* induce plasmacytoid dendritic cells to release interferon, which in turn signals through interferon receptors on other cell types to induce expression of ISGs. This limits the extent to which genetic associations in the population can be integrated with the results of high-throughput experimental perturbations in cell lines.^27^

The ISGs that are genetically downregulated in inflammatory bowel disease are genetically upregulated by the same *trans*-eQTLs in systemic lupus erythematosus.^9^^,^^28^ This indirectly validates the ability to detect effects on interferon signaling through *trans*-effects on expression of ISGs, as the role of genetically upregulated interferon signaling in systemic lupus erythematosus is well-established. The opposite effects of interferon signaling on systemic lupus erythematosus and inflammatory bowel disease are demonstrated by the effects of rare variants in *IFIH1* which encodes a cytosolic sensor of dsRNA that initiates signaling via Type I interferons (interferon-*α* and interferon-*β*) and Type III interferons (interferon-*λ*1 to interferon-*λ*4). Gain-of-function variants in *IFIH1* cause a spectrum of neuroimmunological disorders associated with upregulated Type 1 interferon signaling; loss-of-function variants cause monogenic inflammatory bowel disease.^29^  *IFIH1* is one of 3 genes (*IFIH1*, *RIGI*, *LGP2*) that encode RIG-I like receptors. Variants in *RIGI* are not associated with inflammatory bowel disease in humans, but *Rigi* knockout mice develop spontaneous colitis.^30^

Type I and Type III interferon signaling pathways are regulated by the same sensors of pathogen-associated molecular patterns, and give rise to similar signatures of ISGs. These 2 interferon classes, however, signal through different receptors with different expression profiles. Type I interferons target the interferon-*α∕β* receptor expressed on a broad range of cells. Type III interferons target the IFN-*λ*R1/IL10-R2 (IFN, interferon; IL, interleukin) receptor complex expressed on mucosal epithelial cells. Type II interferon signaling via interferon-*γ* is regulated by cytokines rather than by cytosolic sensors of nucleic acids. Active inflammatory bowel disease is associated with elevated Type II interferon signaling, consistent with the higher levels of interferon-*γ* in cases than in noncases in the UK Biobank proteomics cohort.

Type III interferon signaling maintains host defense against viruses and barrier integrity at mucosal surfaces. In this study, we have suggestive evidence for a specific role of Type III interferon signaling: inflammatory bowel disease was inversely associated with a GATE score for *IFNL1*, which encodes the Type III interferon IFN-*λ*1. More compelling genetic support for a specific role of Type III interferon signaling is a report of 2 patients with very early onset inflammatory bowel disease who were homozygous or compound heterozygous for loss-of-function variants in *IFNL2* and *IFNL3.*^31^ There is experimental support for a protective role of Type III interferon signaling in inflammatory bowel disease. In the mouse model of dextran sodium sulfate-induced colitis, knockout of the interferon regulatory factor *Irf7* or the interferon-*λ* receptor (*Ifnlr1*) gene increased susceptibility to colitis^32^^,^^33^; administration of interferon-*λ2* reversed the effect of *Irf7* knockout.

GATE score associations with inflammatory bowel disease identify 8 other possible core genes, most of which have relatively weak associations with the disease. For 6 of these genes, a role in inflammatory bowel disease is supported by other criteria: GWAS associations with nearby SNPs (*ANKRD55*, *LPP*, *PDLIM7*), experimental perturbation (*CCDC50*, *VCAM1*), association with measured protein levels (*IL6*, *VCAM1*), or drug effects (*IL6*, *VCAM1*). SNPs in *ANKRD55* are associated with multiple immune-mediated inflammatory diseases, but the function of this gene in the immune system is poorly understood.^34^  *CCDC50* encodes a receptor that downregulates the STING-mediated interferon response to dsDNA.^35^  *Ccdc50* knockout in mice reduces the degradation of nucleic acid sensors and promotes interferon signaling.^36^  *PDLIM7* targets the p65 subunit of nuclear factor *κ*B to inhibit inflammatory signaling.^37^ Though *LPP*, which encodes a protein that is localized at sites of cell adhesion, has no obvious specific biological relevance to inflammatory bowel disease, attribution of the nearby GWAS hit to this gene was supported by fine mapping to an intronic SNP.^1^ It is interesting to note that (as shown in Supplementary Table S3) a *trans*-QTL on chromosome 5q for *LPP*, *PDLIM7*, and *VCAM1* contains *CARINH* (colitis-associated IRF1 antisense regulator of intestinal homeostasis, formerly *IRF1-AS1*) that has been shown to protect against colitis in a mouse model by promoting expression of the interferon regulatory factor *Irf1.*^38^

The main limitation of this study is that the genetic prediction of *trans*-effects on gene expression in whole blood relies on eQTLGen Phase I, in which only 10 316 trait-associated SNPs were tested for *trans*-associations. We have shown elsewhere that GATE scores learned from eQTLGen Phase 1 summary statistics explain only about 2% of the variance of measured transcript levels of ISGs in whole blood, though the estimated *trans*-heritability of these transcript levels is about 40%.^6^ This implies that the true size of the effect on inflammatory bowel disease mediated through *trans*-effects on interferon signaling is much larger than the relatively weak effects (standardized odds ratios about 1.05) detected in this study. When summary stats from eQTLGen Phase 2 and other studies of transcriptomics in whole blood become available, the power of GATE analysis to detect core genes and core pathways should be enhanced. Another limitation is that the study relies on transcriptomic studies in whole blood, rather than in the bowel. Direct computation of GATE scores for transcript levels in bowel mucosa would require a collection of consented biopsy samples from at least 30 000 people, which may be technically feasible but is not likely to be established in the near future.

There have been few clinical studies of the relation of Type III interferon signaling to inflammatory bowel disease.^39^ In biopsies taken from uninflamed sites in the terminal ileum or ascending colon, basal expression of the IFN-*λ* receptor subunit (IFN-*λ*R1) and expression of ISGs after ex vivo incubation with IFN-*λ* were reported to be 4- to 7-fold lower in inflammatory bowel disease cases than in controls,^40^^,^^41^ but these results have so far been published only in abstract form. To investigate the role of Type III interferon signaling in inflammatory bowel disease, further experimental and clinical studies are needed. As a model of colitis caused by deficient interferon signaling, the *Rigi* knockout mouse, which develops spontaneous colitis,^30^ may be more relevant than the dextran sodium sulfate-induced model. Deficient Type III interferon signaling may be amenable to therapeutic intervention, for instance, by engineered probiotics^42^ or nutritional supplements.^43^

## Supplementary Material

izaf214_Supplementary_Data

## Data Availability

UK Biobank data are available to approved researchers in the Research Analysis Platform. Summary statistics for expression quantitative trait score analysis were extracted from https://molgenis26.gcc.rug.nl/downloads/eqtlgen/eqts/2019-12-12-eQTSFDR-CohortInfoRemoved-BonferroniAdded.txt.gz
