# Supplementary material for: Genome-Wide Aggregated Trans-Effects Analysis Implicates Deficient Type III Interferon Signaling as a Key Cause of Inflammatory Bowel Disease
Source: Inflamm Bowel Dis. 2025 Sep 25;31(11):3172–82. doi: 10.1093/ibd/izaf214 (PMC12638058; doi:10.1093/ibd/izaf214)
Supplement: izaf214_Supplementary_Data [file izaf214_supplementary_data.pdf]

Supplemental information

Supplementary Tables

Table S1. Replication of associations of eQTL GATE scores with inflammatory bowel disease in Table 1 with associations of transcript levels in whole blood with expression quantitative trait scores (eQTS) for Crohn's or ulcerative colitis

	Heritability of transcript levels		Crohn's		Ulcerative colitis	
Gene	*cis*	*trans*	Z	p-value	Z	p-value
LEF1	0.000	0.46	1.18	0.2	1.72	0.09
ANKRD55	0.194	0.51	1.13	0.3	3.34	8×10^{-4}
CD79B	0.001	0.61	-1.04	0.3	-1.54	0.1
IFIT1	0.022	0.41	-2.13	0.03	-2.53	0.01
IFI44	0.003	0.37	-3.30	0.001	-2.78	0.005
HERC5	0.000	0.39	-1.10	0.3	-3.43	6×10^{-4}
MX1	0.051	0.38	-2.64	0.008	-3.10	0.002
IFI44L	0.000	0.35	-2.87	0.004	-4.01	6×10^{-5}
CCDC50	0.032	0.45	-0.80	0.4	-1.82	0.07

eQTS analysis tests each gene for association of measured levels of expression with a polygenic score for the trait learned from a separate GWAS study of that trait. eQTs summary statistics were extracted from Vosa et al. [9]. Estimates of SNP heritability of transcript levels were extracted from Ouwens et al. [18]

Table S2. Mendelian randomization analysis of effects of putative core genes in Table 1 on disease

Gene	QTL study	Number of QTLs	Estimate of causal effect parameter	p-value
ANKRD55	eQTLGen	13	0.391	4×10^{-5}
VCAM1	UKBPPP	38	0.130	0.003
PDLIM7	UKBPPP	10	-0.494	0.1
CCDC50	eQTLGen	12	-0.355	0.2
LPP	UKBPPP	10	-0.324	0.2
LEF1	eQTLGen	10	0.300	0.3

Tests are restricted to genes with at least 10 *trans*-QTLs. The likelihood of the causal effect parameter is computed by marginalizing over the posterior distribution of the direct (pleiotropic) effects of the genetic instruments on the disease.

Table S3. *Trans*-QTLs for genes identified through eQTL or pQTL effects in Tables 1 or 4.

Chrom	Clump start position (Mb)	Clump end position (Mb)	Target genes	Genes in or near *trans*-QTL clump
1	8.86	8.86	*CCDC50*	.
1	64.75	64.85	*PDLIM7*	*JAK1, RAVER2*
1	158.76	158.81	*LPP*	*OR2AQ1P, OR6N1, OR6N2*
1	160.82	160.82	*LEF1*	*LY9*
1	161.32	161.74	*VCAM1*	*CFAP126*, ***FCGR2A***, ***FCGR2B***, ***FCGR2C***, ***FCGR3A***, ***FCGR3B***, ***FCRLA***, *FCRLB*, ***HSPA6***, *HSPA7, RN7SL466P, RNU6-481P, ...*
1	197.21	197.33	*LPP*	***CRB1***
1	212.71	212.92	*VCAM1*	*FLVCR1, FLVCR1-DT, NSL1, SPATA45, TATDN3*
2	37.73	37.76	*PDLIM7*	*CDC42EP3*
2	43.22	43.58	*LEF1*	*LINC01126, RN7SL531P, RNU6-958P*, ***THADA***, ***ZFP36L2***
2	43.22	43.64	*VCAM1*	*LINC01126, RN7SL531P, RNU6-958P*, ***THADA***, ***ZFP36L2***
2	65.37	65.37	*ANKRD55*	***SPRED2***
2	110.84	111.10	*VCAM1*	*ACOXL, MIR4435-2HG, RPL5P9*
2	110.85	110.86	*LEF1*	*ACOXL*
2	111.11	111.11	*CCDC50*	*ACOXL, ACOXL-AS1, MIR4435-2HG*
2	134.01	135.00	*VCAM1*	*ACMSD, CCNT2, CCNT2-AS1, EDDM3CP, MAP3K19, ...*
2	134.87	135.08	*CCDC50*	*ACMSD, CCNT2, CCNT2-AS1, MAP3K19, RAB3GAP1*
2	135.77	135.77	*CCDC50*	*UBXN4*
2	180.96	181.47	*VCAM1*	***ITGA4***, *LINC01934, MIR4437, UBE2E3, UBE2E3-DT*
2	210.68	210.79	*VCAM1*	*CPS1*
3	46.19	46.26	*LEF1*	***CCR1***, ***CCR3***
3	47.05	47.50	*ANKRD55*	*BOLA2P2, ELP6, KIF9, KIF9-AS1, KLHL18, ...*
3	56.81	56.87	*PDLIM7*	*ARHGEF3*
3	58.22	58.48	*VCAM1*	*ABHD6, HTD2, PDHB, PXK, RPP14*
3	98.62	98.69	*VCAM1*	*WWP1P1*

3	101.55	101.55	*ANKRD55*	.
3	123.11	123.14	*PDLIM7, LPP*	*PDIA5*
3	128.58	128.62	*CCDC50*	.
3	143.38	143.42	*VCAM1*	*SLC9A9, SLC9A9-AS2*
4	38.37	38.37	*LEF1*	*LINC02513*
4	88.89	88.89	*ANKRD55*	*FAM13A*
4	102.51	102.66	*ANKRD55, CCDC50, LEF1*	***MANBA***, ***NFKB1***
4	153.46	153.47	*VCAM1*	*TMEM131L*
5	16.49	16.51	*PDLIM7*	*RETREG1*
5	35.85	35.87	*ANKRD55*	***IL7R***
5	39.42	39.46	*VCAM1*	*DAB2*
5	72.37	72.45	*VCAM1*	*YBX1P5, ZNF366*
5	130.36	132.53	*LPP, PDLIM7, VCAM1*	***ACSL6***, *ACSL6-AS1, ACTBP4, ARL2BPP4,* ***CDC42SE2***, ***CSF2***, ***FNIP1***, ***HINT1***, ***IL3***, ***IRF1***, *IRF1-AS1, LINC02863,* ***LYRM7***, ***P4HA2***, ***PDLIM4***, ***RAPGEF6***, ***SLC22A4***, ***SLC22A5***, ...
5	158.83	158.83	*CD79B*	*EBF1*
6	70.50	70.50	*VCAM1*	*FAM135A*
6	134.61	134.76	*VCAM1*	*LINC03002*
7	17.77	18.00	*VCAM1*	*MRM3P2, SNX13*
7	28.12	28.16	*ANKRD55*	***JAZF1***
7	50.22	50.33	*CCDC50, IFIT1, MX1, HERC5, IFI44, IFI44L*	.
7	106.64	106.74	*PDLIM7, LPP*	*CCDC71L*
7	129.14	129.14	*VCAM1*	.
8	9.09	9.09	*VCAM1*	*ERI1*
8	105.50	105.58	*PDLIM7*	*ZFPM2, ZFPM2-AS1*
8	129.56	129.61	*CD79B*	*CCDC26*
9	0.28	0.31	*LPP*	*DOCK8*
9	111.62	111.63	*VCAM1*	*LRRC37A5P*
9	133.19	134.11	*VCAM1*	*ABO, ADAMTS13, ADAMTSL2, ARF4P1, BRD3, ...*
10	6.05	6.06	*ANKRD55*	***IL2RA***
10	61.96	61.96	*CD79B*	*ARID5B*
10	80.45	80.60	*VCAM1*	*SH2D4B,* ***TSPAN14***, *TSPAN14-AS1*
10	86.25	86.26	*VCAM1*	*GRID1*
10	99.52	99.55	*CCDC50*	*LINC01475,* ***NKX2-3***
11	0.20	0.27	*LPP*	*BET1L, MIR6743, PSMD13, RIC8A, SIRT3*

11	126.35	126.50	*VCAM1*	*DCPS, GSEC, KIRREL3, ST3GAL4*
12	70.70	70.73	*VCAM1*	*PTPRR*
12	103.48	103.84	*VCAM1*	*C12orf42, LINC02401, NT5DC3, STAB2, U8*
12	109.89	112.80	*VCAM1, ANKRD55, CCDC50, CD79B, LEF1, IFI44, IFI44L*	***ACAD10***, *ADAM1A, ADAM1B*, ***ALDH2***, ***ATXN2***, *ATXN2-AS*, ***BRAP***, *CUX2*, ***ERP29***, *HECTD4*, ***MAPKAPK5***, ***NAA25***, ***SH2B3***, ***TMEM116***, ...
12	121.59	122.04	*PDLIM7, LPP*	*BCL7A, CFAP251, HPD, KDM2B-DT, LINC01089, ...*
12	122.33	122.72	*VCAM1*	*CLIP1, CLIP1-AS1, HCAR2, HCAR3, KNTC1, ...*
13	28.03	28.19	*VCAM1*	*CHCHD2P8, FLT3, KATNBL1P1, PAN3, PAN3-AS1*
13	42.38	42.48	*ANKRD55*	*LINC02341*
13	108.34	108.35	*CCDC50*	.
15	42.44	42.56	*VCAM1*	*HAUS2, LRRC57, SNAP23, ZNF106*
15	79.97	79.97	*CCDC50*	*BCL2A1*
15	96.97	96.97	*LPP*	.
16	11.31	11.39	*VCAM1*	***RMI2***
16	30.63	31.12	*ANKRD55*	*BCKDK, BCL7C, CFAP119, CTF1, CTF2P*, ***SETD1A***, ...
16	85.98	85.99	*CD79B*	.
16	89.63	89.87	*VCAM1*	*CDK10, CHMP1A, DPEP1, FANCA, LINC02166, ...*
17	1.56	2.11	*VCAM1*	*DPH1, DPH1-AS1, HIC1, MIR132, MIR212, ...*
17	2.83	2.83	*CD79B*	*RAP1GAP2*
17	7.06	8.03	*VCAM1*	*ACADVL, ACAP1, ASGR1, ASGR2, ATP1B2, ...*
17	29.33	29.56	*LPP, PDLIM7*	*MIR4523, RNU4-34P, RNU6-1034P, RNU6-711P, RPL35AP35, ...*
17	39.66	40.01	*CD79B, LEF1, ANKRD55*	***ERBB2***, ***GRB7***, ***GSDMA***, ***GSDMB***, ***IKZF3***, *KRT8P34*, ***LRRC3C***, *MIEN1, MIR4728*, ***ORMDL3***, *PGAP3*, ***PNMT***, ***STARD3***, ***TCAP***, ***ZPBP2***
17	81.24	81.30	*VCAM1*	*SLC38A10*
19	7.76	7.79	*VCAM1*	*CLEC4GP1, CLEC4M*

19	33.27	33.27	*CD79B*	.
20	32.79	32.79	*ANKRD55*	***DNMT3B***
20	46.07	46.12	*CD79B, LEF1*	***CD40***, *NCOA5, RPL13P2*
20	50.34	50.34	*CCDC50*	.
20	62.41	62.41	*VCAM1*	.
22	30.03	30.20	*LEF1*	*CNN2P1*, ***HORMAD2***, *HORMAD2-AS1*, ***MTMR3***, *RPS3AP51*
22	43.93	44.00	*VCAM1*	*PARVB, PNPLA3, RPL35AP36, SAMM50*

In the clumps of *trans*-eQTLs, genes previously reported as GWAS hits are shown in bold, together with up to 5 genes not reported as GWAS hits. The *trans*-eQTL clump in the HLA region is excluded from this table. *Trans*-QTLs for interferon-stimulated genes are shown separately in Table 1.
